# Production, Characterization of Tannase from *Penicillium montanense* URM 6286 under SSF Using Agroindustrial Wastes, and Application in the Clarification of Grape Juice (*Vitis vinifera* L.)

**DOI:** 10.1155/2014/182025

**Published:** 2014-11-23

**Authors:** Juliana Silva de Lima, Roberta Cruz, Julyanna Cordoville Fonseca, Erika Valente de Medeiros, Marília de Holanda Cavalcanti Maciel, Keila Aparecida Moreira, Cristina Maria de Souza Motta

**Affiliations:** ^1^Department of Mycology, Federal University of Pernambuco, 50670-901 Recife, PE, Brazil; ^2^Unidade Acadêmica Garanhuns, Universidade Federal Rural de Pernambuco, 55292-270 Garanhuns, PE, Brazil

## Abstract

Tannase is an enzyme that hydrolyzes esters and lateral bonds of tannins, such as tannic acid, releasing glucose and gallic acid and stands out in the clarification of wines and juices. Fungi of the genera *Aspergillus* and *Penicillium* are excellent producers of this enzyme. The search for fungi that produce high levels of tannase as well as new substrates for the enzyme production by the SSF is required. The objectives of this study were to evaluate the production of tannase by *Aspergillus* and *Penicillium* species through SSF using leaves and agroindustrial waste barbados cherry and mangaba fruit as substrate, select the best producer, optimize production, characterize the crude enzyme extract, and apply it the clarification of grape juice. Selecting the best producer was performed by planning Placket-Burman and RSM. *P. montanense* showed highest activity with 41.64 U/mL after 72 h of fermentation residue using barbados cherry, with 3.5% tannic acid and 70% moisture. The enzyme showed the highest activity at pH 9.0 and 50°C. The tannase of *P. montanense* was stable over a wide pH range and temperature and, when applied to grape juice, showed higher efficiency by reducing 46% of the tannin content after incubation 120 m.

## 1. Introduction

Tannins are plant constituents, reported as the fourth most abundant group of compounds of these organisms, surpassed only by cellulose, hemicellulose and lignin. They are water soluble polyphenols with molecular mass ranging from 0.3 to 5 kDa [1, 2]. Can be found in many plants and plant residues as* Anacardium occidentale* (cashew),* Vitis vinifera* (grape),* Malpighia glabra* (Barbados cherry), and* Hancornia speciosa* (mangaba fruit) [3, 4]. Such residues, rich in tannins, can be excellent substrates for the production of tannase.

The tannase, also known as tannin acyl hydrolase (EC 3.1.1.20) hydrolyse ester bonds and side hydrolyzable tannins such as tannic acid, gallic acid, and glucose by releasing. Have many applications especially in the beverage industry as beer and wine and instant teas and coffees, as well as in the production of gallic acid and clarification of fruit juice rich in tannins, aiming to reduce the astringency of such products [5]. Such enzymes are naturally produced by ruminant animals, plants, and microorganisms such as filamentous fungi belonging to the genera* Aspergillus* and* Penicillium*. The genus* Aspergillus* is considered as the best producer, followed by* Penicillium*, both standing out as great decomposers of tannins [6].

Industrially the production of tannase is usually performed via submerged fermentation (SF); however, this method can be quite costly for the industry, especially the demand for large quantities of water. Alternatively, to minimize production costs, there is the solid-state fermentation (SSF) [7]. In this type of fermentation medium of production is simple and can be used agroresidue by-products such as bark and seeds of vegetables rich in tannins, plus tannic acid and microorganism grows in the absence of free water [8].

In this context the agroindustrial waste generated by fruit pulp industries, which are very common in tropical countries such as Brazil, are discarded and often become environmental pollutants [8]. An alternative solution to this problem would be the use of such agro-industrial wastes as sources of carbon, nitrogen, and tannins for the production of tannase by microorganisms through SSF.

The objectives of this study were to select the best individual producers of tannase, optimize enzyme production using the best producer, evaluate the production of tannase from* Aspergillus* and* Penicillium* species in the URM Culture Collection maintained by SSF using leaves and barbados cherry and agroindustrial waste mangaba fruit as substrate, and select the best producer of the enzyme, as well as to characterize the crude enzyme extract and apply it in the clarification of grape juice (*Vitis vinifera* L.).

## 2. Materials and Methods

### 2.1. Substrates

Agroindustrial waste barbados cherry (*Malpighia emarginata*) and mangaba fruit (*Hancornia speciosa *Gomez) were obtained from fruit pulp industry, in the municipality of Paulista in Pernambuco, Brazil. The leaves of these fruits were obtained in Jaboatão dos Guararapes in Recife-PE, Brazil. The agroindustrial waste and the leaves were washed with sterile distilled water and dried in an oven at 60°C for 48 hours.

### 2.2. Tannin Estimation

The estimation of tannin content was done following the protein precipitation method [9]. Dried leaves were ground into particles of 50 *μ*m in methanol and kept overnight at 4°C. One mL of extract was taken in a tube and 3 mL of BSA solution was added and kept for 15 min at room temperature. The tubes were centrifuged at 5000 g for 10 min, supernatant was discarded, and pellet was dissolved in 3 mL of SDS-triethanolamine solution. One mL of FeCl_3_ solution was added and tubes were kept for 15 min at room temperature for color stabilization. Color was read at 530 nm against the blank.

### 2.3. Microorganisms and Preparation of Inoculum

15 strains belonging to 15 species of* Aspergillus* and 15 strains belonging to 15 species of* Penicillium* preserved under mineral oil [10], the URM Culture Collection, Department of Mycology, Centre of Biological Sciences, Federal University of Pernambuco, Brazil, were used. These precultures were tested for production of mycotoxins (ochratoxin A and patulin citrinin); no production of such compounds is found (unpublished data).

Each isolate was inoculated on malt extract agar (MEA), contained in a test tube, and incubated at 30°C. After growth, the cultures were maintained at 4°C for short-term use. Then, spores of each culture were transferred to assay containing 10 mL of sterile distilled water and 0.1% Tween 80 pipe. Spore suspension was used as inoculum. The spores were measured by plate count technique at a concentration of 5 × 10^8^ spores/mL [6].

### 2.4. Production of Tannase by Solid State Fermentation (SSF)

Five grams of the residue and of the leaves of barbados cherry (*Malpighia emarginata* DC) and mangaba fruit (*Hancornia speciosa* Gomez) were placed separately in flasks of 250 mL Erlenmeyer flasks and sterilized at 121°C for 30 min in flowing steam. The substrates were moistened with 5 mL of sterile salt solution containing 0.5% w/v of NH_4_NO_3_, 0.1% w/v MgSO_4_·7H_2_O, and 0.1% w/v NaCl, pH 5.0. The moisture content was adjusted to 50%. Each vial was inoculated with 1 mL of spore solution (5 × 10^8^ spores/mL). The contents were mixed and incubated at 30°C for 96 h [6]. After this period, to each bottle was added 50 mL of distilled water containing 0.01% Tween 80, previously sterilized. Then the vials were shaken in a rotary shaker (Tecnal TE421, São Paulo, Brazil) at 150 rpm for 10 minutes. Then the contents were filtered using Whatman number 1 paper filter and the filtrate was regarded as crude enzyme extract and packaged in conical vials and preserved at 4°C for later analysis [6].

### 2.5. Determination of the Activity of Tannase

The tannase activity by spectrophotometry according to the method of Sharma et al. [11] was determined. This method is based on the formation of a chromogen from gallic acid (released by the esterase activity of tannase) and rhodanine (2-thio-4-ketothiazolidine). To determine the gallic acid, 100 *μ*L crude enzymatic extract was incubated with (0.3 mM) tannic acid (10 mM, pH 5.5), sodium phosphate buffer for 30 min at 30°C. Then, 300 *μ*L of the methanolic solution of rhodanine (0.667% w/v rhodanine in 100% methanol) and 100 *μ*L of 500 mM KOH were added to the mixture which was diluted with 860 mL distilled water and incubated for 10 min at 30°C. After this period, samples were read in a spectrophotometer (Hitachi-U5100) absorbance of 520 nm. Standard curve was performed using gallic acid in different concentrations. All assays were performed in triplicate. One unit of tannase activity (U) was defined as the amount of enzyme required to release one micromole of gallic acid per minute under the defined reaction conditions. Enzyme yield was expressed in U/mL [11].

### 2.6. Statistical Analysis

#### 2.6.1. Optimization of the Production of Tannase

Planning Plackett-Burman (PB) and response surface methodology (RSM) design: to optimize the best conditions for the production of tannase from* Penicillium montanense* URM6286, two stages were used.

#### 2.6.2. Identification and Selection of the Most Important Variables in the Optimization Using the Plackett-Burman Design Planning (PB)

For selection of media components to produce tannase, Plackett-Burman (PB) design was used in which the variables were measured: time (h); temperature (°C); moisture (%); pH; tannic acid (%); yeast extract (%); urea (%); glucose (%); starch (%); manganese sulfate (MnSO_4_) (%); monobasic potassium phosphate (KH_2_PO_4_). Each component was examined at two levels: “−1” for low level and “+1” high, using the statistical package Statistica 8.0 software, which generated a set of 12 experimental trials.

#### 2.6.3. Optimization of the Selected Components Using the Response Surface Methodology (RSM)

For knowledge of the optimal level of each of the three variables selected by Plackett-Burman Design Planning (temperature (°C), moisture (%), and tannic acid (%)) was applied the central composite design (CCD) using the STATISTICA 8.0 statistical software. Each variable was assessed in five variables, totaling 20 experimental runs, with six replicated center points. The independent variables were studied at three different levels: low (−1), medium (0), and high (+1) (Table 2). The experiments were conducted in Erlenmeyer flasks of 250 mL containing 5 grams of each residue of the added salt solution (pH 5.0) prepared in accordance with the design, for 72 hours.

### 2.7. Effect of pH and Temperature on the Activity of Tannase

The optimum pH for the activity of tannase present in the crude enzyme extract was determined using different buffers at 0.1 M (citrate phosphate 3.0–6.0, 6.0–8.0 phosphate, Tris-HCl 8.0-9.0, and carbonate-bicarbonate 9.0-10.0) residual activity being determined according to item 2.4 above. Optimum temperature for different temperatures ranging between 30 and 90°C (with intervals of 10°C), using the buffer that expressed the optimum pH for the activity of tannase, was evaluated. The enzymatic activity was estimated in relative activity.

### 2.8. Thermal Stability and pH

The thermal stability of the enzyme was analyzed by incubating the samples at different temperatures ranging from 30 to 90°C for 120 minutes. The pH stability was examined by varying the pH between 3.0 and 10.0 for 2 hours. The residual activity was estimated in the temperature and pH optima.

### 2.9. Application of the Crude Enzyme Extract of Grape Juice for Clarification

For the preparation of juice, fruit grape (*Vitis vinifera* L.) was washed in running water, the seeds were removed and pulp was liquefied (Black & Decker, LF910) and then filtered with the aid of particle size sieve (strainer in stainless steel—ASTM 1/4 inch opening 6.30 mm). The juice was stored at −4°C for further analysis [6]. To clarify juice in vials Erlenmeyers 125 mL were added 10 mL grape juice with different aliquots crude enzymatic extract: (0.5 mL; 1.0; 1.5 and 2.0 mL), which contained 41.54 U/mL activity tannase. The grape juice without crude enzyme extract was used how to control. Then, the vials were placed on a rotary shaker at 150 rpm at a temperature set at 37°C for 120 minutes and analyzed every 30 minutes (0, 30, 60, 90, and 120 min). Assays were performed in five times. After stirring, the flasks were incubated in water bath for 10 min at 50°C. After this period, 1 mL was removed juice treated with crude to determine the concentration of enzyme extract tannins [9].

The tannins content present in the grape was determined by the method of protein precipitation by tannins [9].

With the data of temperature and pH optimum, pH stability and temperature and application were obtained and selected regression lines. Models were tested and were selected based on the coefficient of determination (*r* ^2^) and mean square residual (QMR).

## 3. Results and Discussion

The tannin content of each substrate was estimated by the colorimetric method described by Hagerman and Butler [9]. The highest level of tannins was observed in agroindustrial waste barbados cherry (14.00 mg/g), followed by leaves mangaba fruit (13.11 mg/g), residue mangaba fruit (10.37 mg/g), and leaves of barbados cherry (6.75 mg/g). Although residue barbados cherry has submitted the highest content of tannins, all residues were tested as substrates for fermentation by micro-organisms evaluated.

Bibliographical survey conducted by Aguilar et al. [12] and Belur and Mugeraya [13] between the years 1969 to 2007, it became clear that there are few publications on production of tannase by* Aspergillus* and* Penicillium* species with only nine works cited. Similar survey was conducted in this work showing that there was a small increase in the number of studies using species of* Aspergillus* and* Penicillium* to produce tannase (Table 1).

All 30 strains tested produced tannase when inoculated on the four substrates tested by SSF (Table 2). However, the level of production varied when they were inoculated on different substrates. When incubated in medium containing leaves mangaba fruit the top three producers were* Penicillium digitatum* URM 6216 (27.92 U/mL),* Aspergillus clavatus* URM 5076 (20.14 U/mL), and* Aspergillus avenaceus* URM 5051 (18.57 U/mL). When the residue mangaba fruit (was used as a substrate, were the three best producing* Aspergillus japonicus* URM 5751 (22.85 U/mL),* A. avenaceus* URM 5051 (14.35 U/mL), and* A. parasiticus* URM 5963 (13.35 U/mL). Using barbados cherry leaves as substrate, the top three producers were* Penicillium purpurogenum* URM 6277 (5.92 U/mL),* P. glabrum* URM 6092 (5.78 U/mL), and* P. montanense* URM 6286 (5.42 U/mL). Agroindustrial wast barbados cherry stood out* P. montanense* URM 6286 (31.88 U/mL),* A. terreus* URM 6089 (27.64 U/mL), and* P. purpurogenum* URM 6277 (14.28 U/mL).

15 species of each genus recognized as the best producers of tannase were evaluated. All strains tested showed significant enzymatic activity. However, contradicting reports in the literature is the genus* Penicillium* that stood out and the isolate of* P. montanense* URM 6486 the best producer of the enzyme, with 31.88 U/mL, using barbados cherry residue as substrate. This is the first report of the production of tannase for this species.

The residue best induced production of the enzyme was acerola.* Penicillium Montanense* URM 6486 stood out in the production of tannase using residue barbados cherry, being selected for the optimization of enzyme production.

In 2005, Sabu et al. [6] evaluated the use of tamarind agroindustrial waste, fruit of the tamarind tree (*Tamarindus indica* L.) and palm oil, oil palm fruit (*Elaeis guineensis*) as substrates for the production of tannase by* Aspergillus niger* ATCC 16620. As a result, the authors found that agroindustrial waste oil palm potentiated increased production of the enzymatic activity (13.03 U/g) compared with tamarind residue (6.44 U/g). In the present study, both the WIC and the residue mangaba fruit potentiated the enzyme production by the fungi; however, higher tannase activities were obtained when barbados cherry residue was used as substrate (31.88 U/mL). This is the first study to recycle these agroindustrial wastes for the production of tannase.

To increase the production of tannase, medium components were optimized using the Plackett-Burman experimental design (PB) and response surface methodology (RSM), to select important variables in the production of tannase and verify the significant levels 11 variables were analyzed: time (h); temperature (°C); moisture (%); pH; tannic acid (%); yeast extract (%); urea (%); glucose (%); starch (%); manganese sulfate (MnSO_4_) (%); monobasic potassium phosphate (KH_2_PO_4_) (Table 3) for producing tannase. The effect of each variable and the coefficient is in Table 4 Statistically significant variables at a confidence level of 95% were temperature (°C), moisture (%), and tannic acid (%) (Table 4). All effects of the significant variables were positive, which shows that the influence of these variables was greater in larger parameters tested in the production of tannase from* Penicillium montanense* URM 6286.

The temperature and tannic acid have been identified as most significant variables for the production of tannase by* Penicillium montanense* URM 6286 in SSF using Plackett-Burman, followed moisture model variable (Table 4). These variables were selected to be optimized using parameters supplied by the statistical program, the central composite design (CCD) (Table 5).

After the completion of the Plackett-Burman design (PB), temperature and moisture content and percentage of tannic acid were identified as significant variables. According to the PB, the highest temperature (36°C) allowed greater activity. However, after application of the response surface methodology (RSM) was observed that 34°C is ideal for the production of tannase by* P. montanense* URM 6486. Besides the best temperature, the RSM identified the best concentration of tannic acid (3.5%) for the fungus under study produces high level of tannase. The results of this study are corroborated with those found by Seth and Chand [14]. These authors showed increased of the production of tannase by Aspergillus awamori in the presence of 3.5% tannic acid.

Data were subjected to analysis of variance (ANOVA), with the levels of production of tannase and the experimental and predicted data presented in Table 4. Quadratic regression equation was best explained the optimization of environmental variables to produce tannase with an *r* ^2^ of 0.74 explaining 74% of the variability of the model and showing the quality of the model.

The interactions between the variables generated three-dimensional graphs showing the increase in the production of tannase, suggesting a great production (41.64 U/mL) in culture medium moistened at 70%, plus 3.5% tannic acid, and incubated at 34°C. Therefore, there was an increase of approximately 26% in the production of the enzyme compared to the maximum activity obtained in planning PB (32.92 U/mL) and approximately 30% compared to the initial screening, proving the validity of the model optimization.

**Figure 1:**
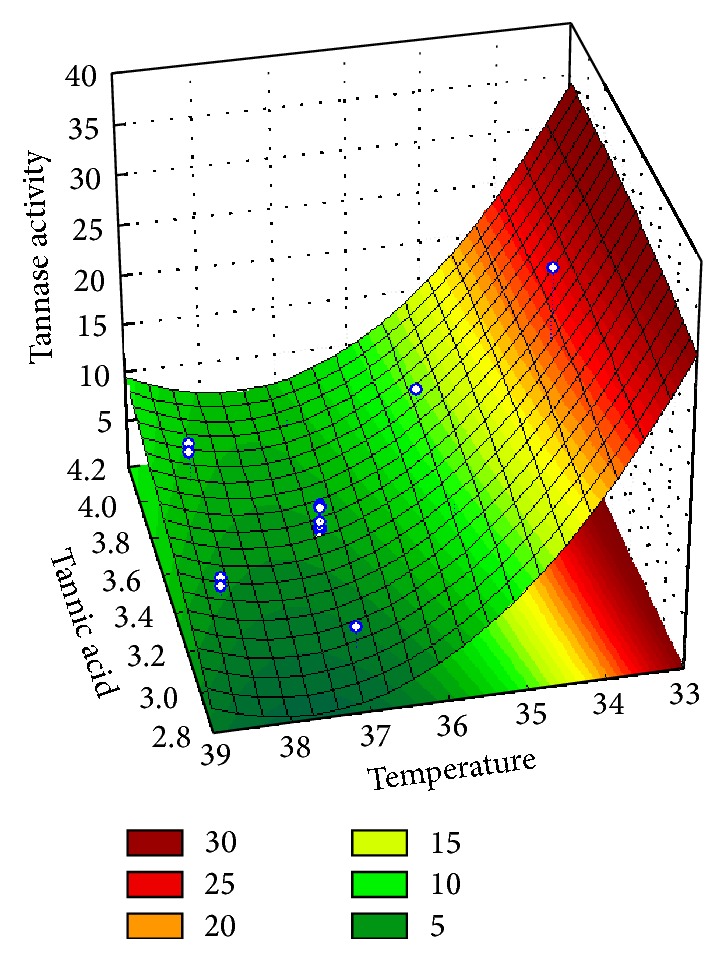
Response surface graph showing the effect to tannic acid and temperature interaction on tannase activity in SSF.

**Figure 2:**
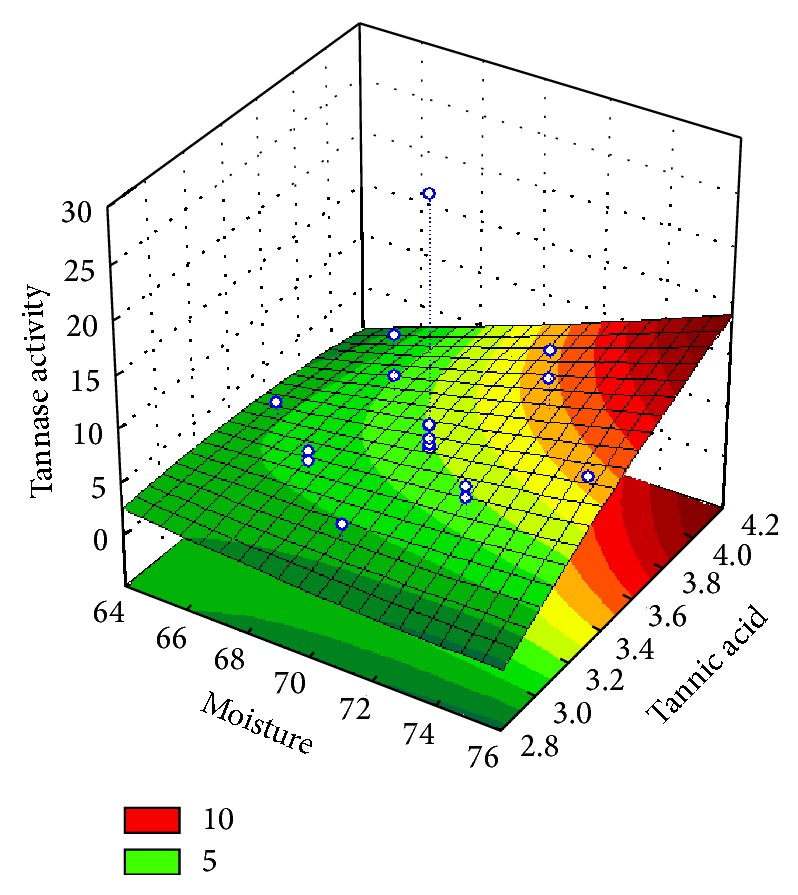
Response surface graph showing the effect of tannic acid and moisture interaction on tannase activity in SSF.

With the planning of RSM when analyzed simultaneously and temperature factors tannic acid content of agreement, we observed that tannase production by* P. montanense* URM 6486 increases with increasing content of tannic acid and when the temperature of incubation decreases (Figure 1). Also according the RSM when the factors moisture content and concentration of tannic acid are simultaneously analyzed, it is observed that the higher the moisture and the concentration of tannic acid (Figure 2), the greater the production of tannase by the microorganism study.

In recent years, some studies on the production of fungal tannase through SSF are used as substrates agro agroindustrial waste like leaves of vegetables rich in tannins [5, 15, 16]. Kumar et al. [15], for example, evaluated the use of leaves of jameloeira (*Syzygium cumini*), tropical tree having leaves rich in tannins, as a substrate for the production of tannase from* Aspergillus ruber*. In this study, the authors obtained 69 U/g as maximum tannase activity after 96 hours test at 30°C and pH 5.5. Yadav et al. [16] evaluated different fruit trees leaves (*Phyllanthus emblica, Zizyphus mauritiana, Diospyros montana, Prosopis juliflora,* and* Syzygium cumini*), as substrates for production of tannase by* Aspergillus fumigatus*. According to the authors, the best parameters were established for production of tannase through the SSF. In this case, the leaves of* Syzygium cumini* were presented as the best substrates for enzyme production. After optimization of the process, the maximum production of tannase of 174.32 U/g was obtained with 96 h at 25°C and pH 5.

M. K. Selwal and K. K. Selwal [5], in turn, evaluated tannase production by* Penicillium atramentosum* KM isolated from tannery effluents. Leaves of* Phyllanthus emblica*,* Zizyphus mauritiana*,* Diospyros montana*,* Syzygium cumini,* and* Prosopis juliflora* were used as substrates. The fermentation tests were carried out at 30°C for 72 h. According to the authors, the best substrates were* Prosopis juliflora* leaves, which showed a maximum tannase activity of 34.7 U/mL, and the leaves of* Phyllanthus emblica* showed a maximum tannase activity of 32.8 U/mL. In the present study, when the leaves mangaba fruit were used as substrate, they showed more significant results than the barbados cherry because potentiated by higher activities of tannase tested strains, especially* P. digitatum* URM 6216 (27.92 U/mL). These published data show that the use of agro and agro agroindustrial wastes as substrates for the production of tannase potentiate the production of this enzyme by microorganisms evaluated, important factors for the success of a high enzyme production also minimizing production costs.

**Figure 3:**
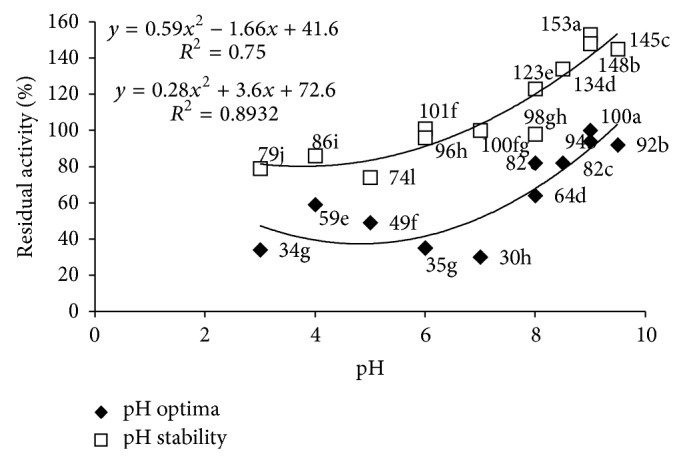
Effect of pH and stability (2 hours incubation) on the activity of tannase from* Penicillium montanense* URM 6486.

Regarding the optimum pH, the higher tannase activity was obtained at pH 9.0 in carbonate-bicarbonate buffer with 100% residual activity and explained by polynomial curve with *r* ^2^ = 0.75 and 0.89. Regarding the stability of tannase produced by* P. montanense* URM 6486 throughout the pH range tested the enzyme showed more than 70% activity. Excelling at pH 9.0 in Tris-HCl buffer enzyme showed 153% residual activity. These results show the tannase* P. montanense* 6486 URM is stable over the entire pH range (Figure 3).

Both the pH, the initial temperature of the culture medium is of great importance in the production of metabolites [5]. According to Belur and Mugeraya [13], the pH optimum for the production of fungal tannase by means SSF is between the range of 5.0–6.5 and the fermentation time varies from 72 to 86 h. In the present study, the highest activity was found in culture medium with initial pH 5.0 for 72 h of incubation, as reported in the literature [17]. For the industry producing enzymes, it is important that the microorganism can maximum production in a relatively short time, a factor that minimizes production costs. To this* P. montanense* URM 6486, it is extremely interesting, because it stood out as a great producer of the enzyme.

M. K. Selwal and K. K. Selwal [5] first reported as* Paecilomyces varioti* isolated fungal tannase producing at high pH (7.5), by using SSF keekar sheets (*Acacia nilotica*). In the present study,* P. montanense* URM 6486 was able to produce tannase also by SSF using residue barbados cherry, in extremely basic pH (9.0), stable over a wide pH range, overcoming the results obtained by M. K. Selwal and K. K. Selwal [5]. This feature is important because it minimizes costs dispensed in pH control during the manufacturing process.

**Figure 4:**
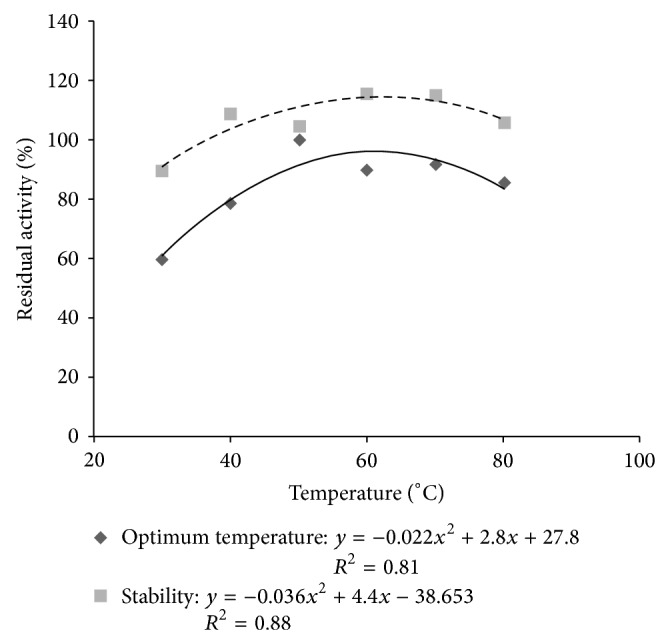
Effect of temperature and stability (2 hours incubation) on the tannase activity.

The effect of temperature on the activity of tannase showed the maximum activity was obtained at 50°C with 100% residual activity. The enzyme was stable at all temperatures tested with over 80% residual activity. These results show the tannase* P. montanense *URM 6486 is stable over the entire temperature range (Figure 4).

The effect and temperature stability (2 hours of incubation) on the activity of tannase* Penicillium montanense* 6486 URM are best explained by polynomial curve, with *r* ^2^ = 0.81 and 0.88, respectively (Figure 4). By the equation, it can be seen that the optimum temperature for the activity of tannase was at 61.38°C, with residual activity of 96.34%.

Another extremely relevant factor to be considered is the effect of temperature on enzyme activity and enzyme stability at temperatures [13]. In this study, the effect of temperature on the activity of tannase showed the maximum activity was obtained at 50°C with 100% residual activity, as well as being stable over a wide temperature range and can be produced by industry at a temperature next room temperature, also minimizing costs.

**Figure 5:**
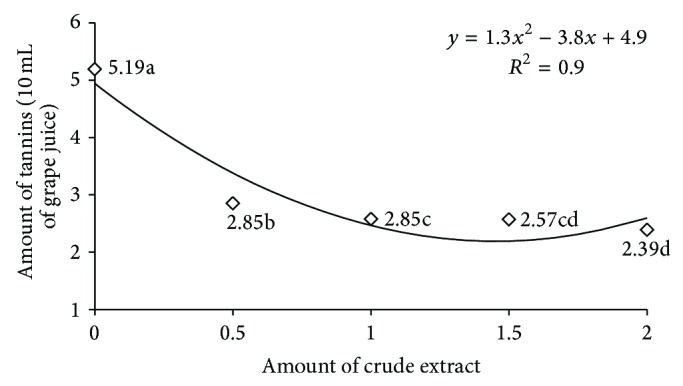
Effect of different volumes of tannase in the degradation of tannins present in grape juice after 120 minutes of incubation. Values on *Y*-axis represent the amount of tannins in the juice.

**Figure 6:**
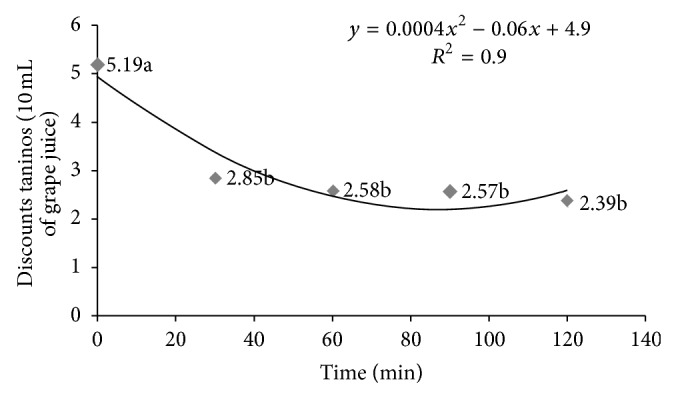
Effect of 2 mL tannase on degradation present in grape juice at different times. Values on the *Y*-axis represent the amount of tannins in the juice.

The crude enzymatic extract of* P. montanense* URM 6486 containing tannase is shown in Figures 5 and 6. Initially juice contained 5.19 mg/g tannins. Clarification was tested in different concentrations of crude extracts (Figure 5), as well as different time intervals (Figure 6). These data were subjected to regression analysis, being selected polimoniais quadratic equations, with *r* ^2^ = 0.9 and statistical analysis, indicating a good quality of the equation to explain the effect. A further increase in the volume of crude enzyme extract (2 mL) improved point of clarification, because, according to the prediction of the equation, the maximum point was with a volume of 2.0 mL crude extract of tannase, with the minimum quantity of tannin, which was 1.44 tannins/10 mL of grape juice (Figure 5) in 86.96 minutes (Figure 6), but no statistically significant difference when applying 1 mL, 1.5 mL, and 2.0 mL. The tannin content in the juice was reduced to 46% (2.39 mg/g) after 2 hours of incubation with the crude enzyme extract at 37°C.

Among the various applications of tannase highlights the possibility of clarification of juices by decreasing the content of tannins present in these products. However, studies related to this application are scarce. In 2006, Rout and Banerjee [18] tested the tannase coproduced by* Aspergillus* and* Rhizopus oryzae* foetidus through SSF in the clarification of pomegranate juice (*Punica granatum*), rich in tannins. The authors observed 25% reduction in tannin content present in the juice by applying 1 mL (35.6 U/mL), after 120 minutes of incubation with the enzyme at 37°C. In the present study, the tannase produced by* P. montanense* URM 6486 (41.64 U/mL), contained in 1 mL of the crude enzymatic extract when applied to grape juice showed higher efficiency by reducing 46% of the content of tannins in the juice after 120 minutes incubation.

In a recent study, Sharma et al. [19] tested the activity of tannase produced by* Aspergillus niger* in detannification of guava (*Psidium guajava*) using 2% of the enzyme in the juice after 60 minutes the authors observed a reduction of 59.23% of tannin content in the juice. Both this result as that obtained in the present study demonstrated the importance of fungal tannase the clarification of juices rich in tannins and may minimize production costs by the industry.

## 4. Conclusions

*Penicillium montanense* URM 6486 is being reported for the first time as a producer of high levels of tannase by SSF using barbados cherry agroindustrial waste as substrate. These residues are also a new substrate for the production of this enzyme high industrial relevance, especially for the food industry. However, both the optimization of the growth parameters in a bioreactor and the purification of the enzyme are necessary to determine the commercial viability of the enzyme and possible application in the clarification of juices like grape.

## Figures and Tables

**Table 1:** Species of *Aspergillus* and *Penicillium* reported as producing tannase between the years 1968 and 2014.

Species	References
*Aspergillus flavus *	[20]
*A. awamore, A. Niger, A. oryzae *	[21]
*A. gallonyces *	[22]
*A. niger *	[23]
*A. fumigatus, A. versicolor *	[24]
*Aspergillus ruber *	[25]
*Aspergillus tamarii *	[26]
*Aspergillus tamarii *IMI388810	[27]
*A. niger *	[28]
*A. niger *	[19]
*Penicillium notatum, P. islandicum *	[29]
*P. chrysogenum, P. digitatum, P. acrellanum,* *P. carylophilum, P. citrinum e P. charlessi *	[21]
*P. glaucum *	[30]
*P. variable, P. crustosum, P. Restrictum *	[24]
*P. glabrum *	[31]
*Penicillium spp. *	[32]
*P. variable *	[33]
*Penicillium canescens, P. zacinthae, P. purpurogenum,* *P. spinulosum, P. frequentans e P. chrysogenum *	[34]
*P. atramentosum KM *	[5]
*Penicillium sp. *EZ-ZH190	[35]

**Table 2:** Tannase activity (U/mL) strains of *Aspergillus* and *Penicillium *produced under solid state fermentation (SSF) using agroindustrial waste and leaves of barbardos cherry (*Malpighia emarginata*) and residue and leaves of mangaba fruit (*Hancornia speciosa* Gomez) as substrates after 96 hours of fermentation.

Lineage	No. URM	Mangaba leaf	Acerola leaf	Residue mangaba	Residue acerola
*Aspergillus aculeatus* Iizuka	4953	8.14	4.14	10.21	4.71
*A. avenaceus *G. Sm.	5051	18.57	5.21	14.35	7.42
*A. carbonarius *(Bainier) Thom	5012	9.35	4.35	10.07	4.35
*A. clavatus *Desm.	5076	20.14	4.57	12.28	6.00
*A. fumigatus* Fresen.	6151	9.57	4.92	9.57	4.78
*A. granulosus* Raper & Thom	4641	15.35	2.92	8.92	5.28
*A. japonicus *Saito	5751	5.00	3.78	22.85	6.28
*A. niger *Tiegh.	5996	6.50	4.14	12.78	4.35
*A. ochraceus* G. Wilh.	5836	12.21	4.21	9.00	10.21
*A. parasiticus* Speare	5963	13.85	4.00	13.35	7.92
*A. tamari *Kita	4634	13.64	5.00	12.00	4.85
*A. terreus *Thom	6089	9.28	5.32	9.71	27.64
*A. ustus *(Bainier) Thom & Church	3842	8.42	5.32	9.35	6.42
*A. viridinutans *Ducker & Thrower	6160	9.71	4.14	7.85	27.00
*A. versicolor *(Vuill.) Tirab.	5000	11.78	4.42	11.28	18.00
*Penicillium aurantiogriseum *Dierckx	6026	4.85	5.07	11.85	4.42
*P. citrinum *Sopp	6286	4.92	3.64	10.92	6.64
*P. commune* Thom	6147	5.42	4.92	12.00	7.21
*P. corylophilum* Dierckx	5967	8.64	4.50	11.35	5.35
*P. digitatum *(Pers.) Sacc.	6216	27.92	4.00	11.21	5.35
*P. fellutanum* Biourge	6137	8.00	3.71	11.28	7.85
*P. glabrum* Wehmer	6092	7.50	5.78	10.78	7.35
*P. lanosum *Westling	6288	4.50	4.35	9.78	3.57
*P. lapidosum* Raper & Fennell	6042	9.14	5.00	11.64	17.50
*P. lividum* Westling	6090	4.85	3.35	10.07	4.42
*P. montanense* M. Chr. & Backus	6286	4.78	5.42	10.14	31.88
*P. purpurogenum* Flerov	6277	5.92	5.92	11.71	14.28
*P. simplicissimum* (Oudem.) Thom	6138	5.71	4.28	12.57	9.78
*P. verruculosum* Peyronel	6222	9.50	5.21	10.42	8.07

**Table 3:** Experimental matrix Plackett-Burman planning (PB) for the production of tannase from *Penicillium montanense* URM 6286 under FES using agroindustrial wastes.

Trials	Time	Temperature	Moisture	pH	Tannic acid	Yeast extract	Urea	Glucose	Starch	Manganese sulfate	Monobasic potassium phosphate
1	120 (+)	36 (+)	35 (−)	8 (+)	0.5 (−)	0.25 (−)	0.25 (−)	0.5 (+)	0.5 (+)	0.1 (+)	0.1 (−)
2	120 (+)	36 (+)	35 (−)	8 (+)	2 (+)	0.25 (−)	0.5 (+)	0.1 (−)	0.1 (−)	0.05 (−)	0.5 (+)
3	72 (−)	36 (+)	65 (+)	5 (−)	2 (+)	0.25 (−)	0.25 (−)	0.1 (−)	0.5 (+)	0.1 (+)	0.5 (+)
4	72 (−)	28 (−)	65 (+)	8 (+)	2 (+)	0.25 (−)	0.5 (+)	0.5 (+)	0.1 (−)	0.1 (+)	0.1 (−)
5	120 (+)	36 (+)	65 (+)	5 (−)	2 (+)	0.5 (+)	0.25 (−)	0.5 (+)	0.1 (−)	0.05 (−)	0.1 (−)
6	72 (−)	28 (−)	35 (−)	5 (−)	0.5 (−)	0.25 (−)	0.25 (−)	0.1 (−)	0.1 (−)	0.05 (−)	0.1 (−)
7	120 (+)	28 (−)	65 (+)	5 (−)	0.5 (−)	0.25 (−)	0.5 (+)	0.5 (+)	0.5 (+)	0.05 (−)	0.5 (+)
8	72 (−)	28 (−)	35 (−)	8 (+)	2 (+)	0.5 (+)	0.25 (−)	0.5 (+)	0.5 (+)	0.05 (−)	0.5 (+)
9	120 (+)	28 (−)	65 (+)	8 (+)	0.5 (−)	0.5 (+)	0.25 (−)	0.1 (−)	0.1 (−)	0.1 (+)	0.5 (+)
10	72 (−)	36 (+)	65 (+)	8 (+)	0.5 (−)	0.5 (+)	0.5 (+)	0.1 (−)	0.5 (+)	0.05 (−)	0.1 (−)
11	72 (−)	36 (+)	35 (−)	5 (−)	0.5 (−)	0.5 (+)	0.5 (+)	0.5 (+)	0.1 (−)	0.1 (+)	0.5 (+)
12	120 (+)	28 (−)	35 (−)	5 (−)	2 (+)	0.5 (+)	0.5 (+)	0.1 (−)	0.5 (+)	0.1 (+)	0.1 (−)

**Table 4:** Results and coefficient effect for the production of tannase presented by the variables used in the Plackett-Burman design (PB).

Variables	Effect	Coefficient
(1) Time	−0.67538	−0.337691
(2) Temperature	3.75272^*^	1.876362
(3) Moisture	1.96078^*^	0.980392
(4) pH	−1.90632	−0.953159
(5) Tannic acid	3.15359^*^	1.576797
(6) Yeast extract	−0.49564	−0.247821
(7) Urea	−1.90632	−0.953159
(8) Glucose	−1.78105	−0.890523
(9) Starch	0.50654	0.253268
(10) Manganese sulfate	−0.43573	−0.217865
(11) Monobasic potassium phosphate	0.96405	0.482026

^*^Significant effect.

**Table 5:** Significant variables used in the response surface methodology (RSM).

Independent variables	−*α*	−1	0	+1	+*α*
Temperature	34	35.5	37	38.5	40
Moisture	65	67.5	70	72.5	75
Tannic acid	3.0	3.25	3.5	3.75	4.0
